# The Role of Ion Channels in Microglial Activation and Proliferation – A Complex Interplay between Ligand-Gated Ion Channels, K^+^ Channels, and Intracellular Ca^2+^

**DOI:** 10.3389/fimmu.2015.00497

**Published:** 2015-10-22

**Authors:** Martin James Stebbing, Jennifer Marie Cottee, Indrajeetsinh Rana

**Affiliations:** ^1^Health Innovations Research Institute and School of Medical Sciences, RMIT University, Bundoora, VIC, Australia; ^2^School of Health Sciences, Federation University Australia, Ballarat, VIC, Australia

**Keywords:** microglia, P2X receptors, ion channels, K^+^ channels, neuroinflammation

## Abstract

Microglia are often referred to as the immune cells of the brain. They are most definitely involved in immune responses to invading pathogens and inflammatory responses to tissue damage. However, recent results suggest microglia are vital for normal functioning of the brain. Neuroinflammation, as well as more subtle changes, in microglial function has been implicated in the pathogenesis of many brain diseases and disorders. Upon sensing alterations in their local environment, microglia change their shape and release factors that can modify the excitability of surrounding neurons. During neuroinflammation, microglia proliferate and release NO, reactive oxygen species, cytokines and chemokines. If inflammation resolves then their numbers normalize again via apoptosis. Microglia express a wide array of ion channels and different types are implicated in all of the cellular processes listed above. Modulation of microglial ion channels has shown great promise as a therapeutic strategy in several brain disorders. In this review, we discuss recent advances in our knowledge of microglial ion channels and their roles in responses of microglia to changes in the extracellular milieu.

## Microglia Express a Variety of Ion Channels

For cells that were, until relatively recently, thought to be quiescent in the normal brain and simply surveilling the surrounding tissue for damage or infection, microglia are exquisitely sensitive to their environment and express a remarkable array of different ion channels. In addition to many neurotransmitter sensitive and other ligand-gated ion channels, there is evidence for the presence of H^+^ channels, Na^+^ channels, voltage-gated Ca^2+^ channels, Ca^2+^-release-activated Ca^2+^ channels, voltage-dependent and voltage-independent Cl^−^ channels, and at least six different types of K^+^ channels in microglia (1). The later include inward rectifier, delayed rectifier, HERG-like, G-protein-activated, as well as voltage-dependent and voltage-independent Ca^2+^-activated K^+^ channels. Some of these channels are species specific, whereas others are commonly expressed between species, but the majority of these appear to be expressed in human microglia (1). For cells that not “excitable” in the neuronal sense, this large array of different channel types channels might seem superfluous, yet a substantial body of evidence suggests these channels regulate all of the important functions of microglia, including activation, chemotaxis, secretion, proliferation, the respiratory burst (2, 3), and phagocytic activity (4). In addition, the expression level of most of these ion channels depends on the functional state of the microglial cell and several have been proposed as therapeutic targets in neurological diseases.

## Ligand-Gated Ion Channels Help Microglia to Monitor Their Local Environment

Microglia express a variety of receptors that detect changes in their local environment, many of which are ligand-gated ion channels, including purinergic receptors, glutamate receptors, and other neurotransmitter gated channels (5–14). For example, microglial glutamate receptors include ionotropic receptors *N*-methyl-d-aspartic acid (NMDA), (S)-a-amino-3-hydroxy-5-methyl-4-isoxazolepropionic acid (AMPA), and kainic acid (KA) receptors in addition to metabotropic glutamate receptors. Many of these receptors are sensitive to microglial secretions acting in an autocrine manner. For instance, neurotransmitters can induce microglial glutamate release, which can then act back on microglia to induce more neurotransmitter release. Microglia express receptors for ATP and cultured microglia release ATP following application of glutamate. This release is prevented by AMPA receptor blockers but not NMDA receptor inhibitors and is dependent on PKC activation and release of Ca^2+^ from stores, but not on extracellular Ca^2+^ (15). This type of autocrine action of microglial secretions has been reported by various *in vitro* studies (8, 11, 15, 16) and it may provide positive feedback to allow rapid responses to danger signals and recruitment of surrounding microglia *in vivo*. However, there is also evidence that microglia express only some of these receptors in their resting condition. Upon activation, they increase their expression of some receptors and begin to express others *de novo*. For instance, microglial P2X4 purinoreceptor expression is increased both by lipopolysaccharide (LPS) treatment *in vitro* (17) and in spinal microglia following a peripheral nerve injury *in vivo* (5).

Among microglial receptors, P2X purinergic receptors have been extensively investigated due to their reported roles in microglial activation in various pathological conditions linked to injury and inflammation (5, 18–20). As distinct from P2Y receptors that are G-protein coupled, P2X receptors are cation-permeable ligand-gated ion channels that open in response to the binding of extracellular purines, such as ATP, which are thought to be released from damaged/degenerating neuronal tissue. In microglial cells, activation of these receptors results in entry of Ca^2+^, which subsequently causes various cellular responses associated with microglial function both *in vitro* and *in vivo*. For instance, several studies suggest a vital role for microglial P2X receptors (5, 21) in generating neuropathic pain following nerve injuries, although microglial P2Y receptors may also be involved (22, 23). Some reports have suggested involvement of P2X receptors in microglial cytokine release and P2Y receptors in microglial chemotaxis (24). As with other immune cells, P2X7 receptors have been implicated not only in microglial inflammatory responses and inflammasome activation (25) but also in microglial proliferation (26, 27). Genetic variants of P2X receptors are associated with neurodegenerative disease and appear to also play a role in microglial phagocytosis (28).

One of the most interesting consequences of microglial P2X receptor activation is the formation of membrane pores large enough to admit molecules, such as NMDG^+^ and DNA binding dyes. This process has been studied extensively for P2X7 receptors and may result in pro-inflammatory actions and cytolysis, but is also apparently required for the proliferative actions of P2X7 in microglia (26). A similar process was recently reported to occur following P2X4 receptor activation in microglial cells without consequent cell death (29). The formation of these pores may enable the release of factors that have paracrine and autocrine functions, such as glutamate (30), but will also pass Ca^2+^ and lead to a sustained increase in intracellular Ca^2+^, which in turn may underlie many of the downstream effects of P2X receptor activation on microglial function.

## Role of Increased Intracellular Ca^2+^ in Microglial Function

**Figure 1:**
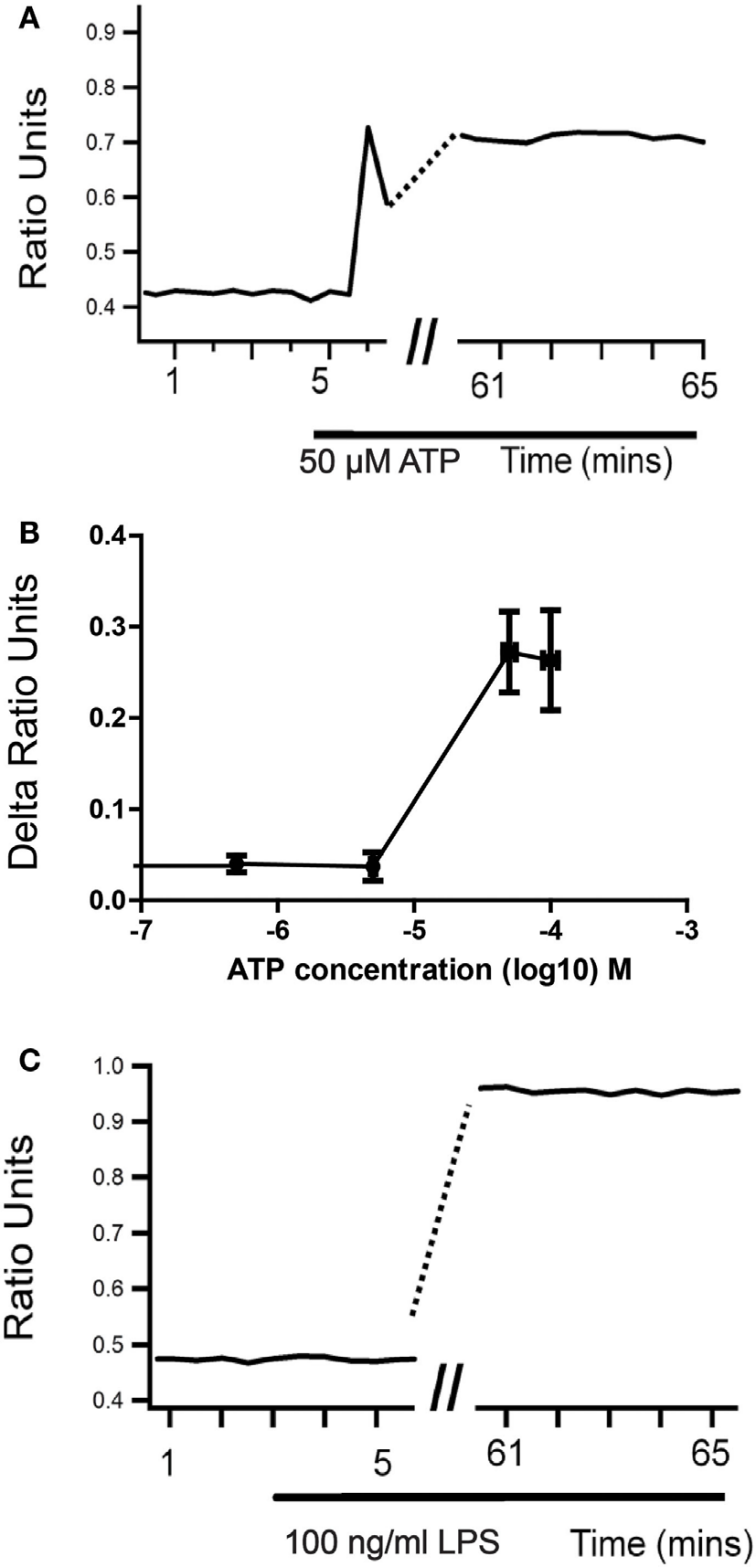
**Sustained changes in intracellular Ca^2+^ concentration in microglia upon activation**. When cultured neonatal rat microglia are stimulated with either ATP **(A,B)** or LPS **(C)**, there is a sustained changed in intracellular Ca^2+^ levels as measured using Fura-2 ratiometric Ca^2+^ imaging that develops over 1 hour.

Microglial responses, including morphological changes, migration, proliferation, and secretion of cytokines and reactive oxygen species, are usually associated with an increase in intracellular Ca^2+^ concentration ([Ca^2+^]_i_) (31, 32). The majority of receptor agonists known to cause microglial activation also cause an increase in [Ca^2+^]_i_, although often via different mechanisms and with markedly different consequences. For example, both ATP and LPS are capable of activating microglia *in vitro* and *in vivo* (33–37) and both cause an increase in [Ca^2+^]_i_. LPS does not produce an immediate Ca^2+^ response, but is reported to cause a sustained increase in basal [Ca^2+^]_i_ in microglia after a 24-h treatment (38), although this increase is evident as early as 1 h after LPS application (Figure 1). An intracellular Ca^2+^ chelator prevented the LPS-stimulated increase in microglial NO, cytokines and chemokine release, but the Ca^2+^ ionophore ionomycin caused none of these effects. This suggests that elevated [Ca^2+^]_i_ is necessary but not sufficient for the pro-inflammatory actions of LPS on microglia (38). By contrast, ionomycin can mimic the stimulation of c-fos expression in microglia via glutamate receptor-mediated calcium influx (13).

Unlike LPS, Ca^2+^ imaging experiments show that ATP induces an immediate, transient increase in [Ca^2+^]_i_ in microglia that is biphasic in many cells. The amplitude of this response is dose dependent and maximal at 300 μM. However, when ATP is applied in the absence of extracellular Ca^2+^, microglia commonly show no response or a smaller monophasic increase in [Ca^2+^]_i_ (39). In the presence of extracellular Ca^2+^ and thapsigargin to block the endoplasmic reticulum calcium pump, microglia either responded to ATP or displayed an increased basal calcium (39), which has been shown to prevent ATP responses (38, 39). These results support the involvement of ionotropic receptors in the response to ATP, although metabotropic receptors and Ca^2+^ induced Ca^2+^ release may also be involved. These responses to ATP are not blocked by 100 μM suramin and similar [Ca^2+^]_i_ increases are seen in response to the selective P2X receptor agonist 2-methylthio ATP, but not αβ-methylene ATP (40) suggesting P2X4 or P2X7 but not P2X1 and P2X3 are involved (41, 42). Although the concentration dependence of the ATP-induced Ca^2+^ influx would suggest mediation by P2X4 receptors, the fact that similar responses are seen to the selective P2X7 receptor agonist 2′- and 3′-*O*-(4-benzoylbenzoyl) adenosine 5′-triphosphate (BzATP) indicates that functional P2X7 receptors are also present. Interestingly, pretreatment with oxidized ATP (oATP; 100 μM), which blocks P2X7 but not P2X4-mediated currents in microglia (43) prevented the increase in [Ca^2+^]i induced by ATP concentrations (10 and 100 μM) that activate P2X4 but not P2X7 receptors (40). One possible explanation for this might be autocrine release of ATP (44), although other actions of oATP cannot be discounted.

A later more sustained increase in [Ca^2+^]_i_ similar to that reported following exposure to LPS can be seen in microglia following prolonged exposure to ATP at 30 μM or more (Figure 1). The time course and concentration dependence of this response are consistent with those of macropore formation in response to P2X4 activation measured via dye uptake (29). In contrast to LPS, however, application of 30–100 μM ATP does not cause release of cytokines, NO or activate a respiratory burst in microglia, again emphasizing that an increase in [Ca^2+^]_i_ is not sufficient to stimulate these pro-inflammatory actions. Indeed, there is evidence that a prior sustained increase in [Ca^2+^]_i_ can inhibit responses of microglia to further stimulation (38, 39). Changes in microglial morphology are seen when microglia are treated with 50–100 μM ATP (45); however, these changes are more subtle that those seen in response to LPS. The activation state induced in microglia by LPS is widely believed to be analogous to the M1 or pro-inflammatory activation state seen in macrophages. Whether or not the state induced by low micromolar concentrations of ATP in microglia is analogous to the M2, “alternate” or anti-inflammatory activation state of macrophages is less certain. It is clear, however, that P2X4 receptors on microglia and their activation by ATP can contribute to neuronal hyperexcitability and pathological changes within the nervous system (5, 40). Higher concentrations of ATP that would activate P2X7 receptors are reported to cause pro-inflammatory actions, such as generation of a respiratory burst (46), but this is not the case in all studies, and may depend on the conditions of the experiment.

**Figure 2:**
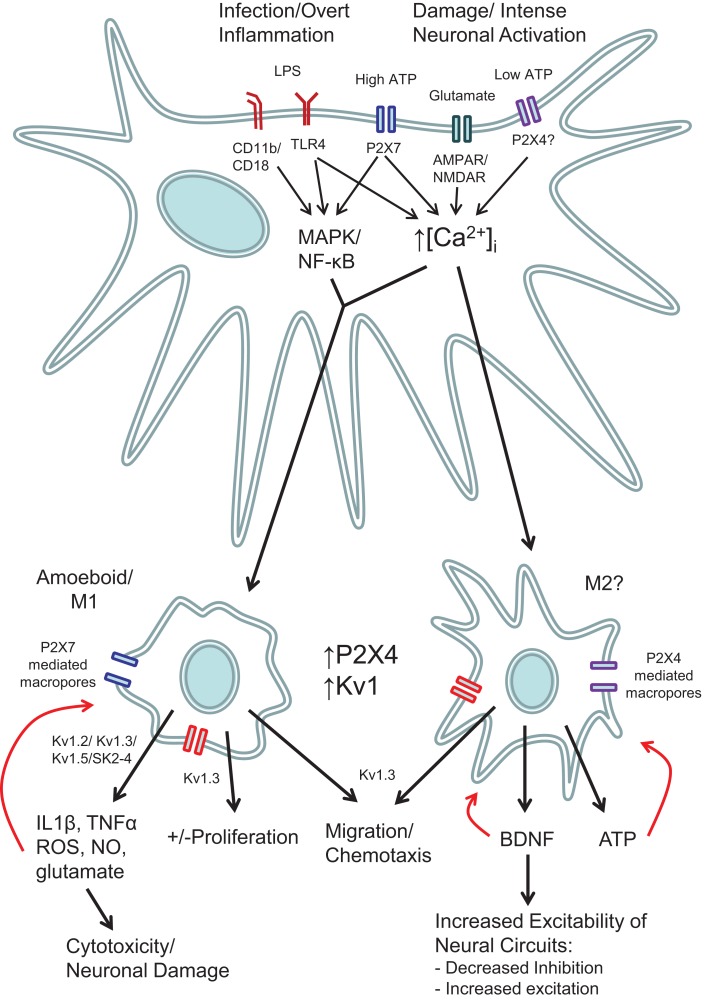
**Schematic showing the various roles of ion channels in microglial function**. Stimulation of microglia with either LPS or high concentrations of ATP (e.g., 3 mM) act via increased intracellular Ca^2+^ and intracellular signaling pathways such as MAP kinases and NF-κB. Low concentrations of ATP that stimulate P2X4 receptors (e.g., 100 μM), do not cause cytokine release or ROS production, but nevertheless do increase intracellular Ca^2+^ and produce morphological and functional changes that can lead to pathological changes in the function of surrounding neurons. Red arrows indicate potential autocrine and positive feedback mechanisms.

Other agents known to increase [Ca^2+^]_i_ in microglia include glutamate (15), complement (38), interferon gamma and BDNF (47). BDNF release from microglia has been implicated in causing the increased excitability seen in dorsal horn neurons following peripheral nerve injury (48, 49). BDNF can also act back on microglial TrkB receptors to induce a sustained elevation of [Ca^2+^]_i_ via a complex mechanism, but BDNF also suppresses IFN-γ-induced calcium influx and the associated increase in iNOS expression and nitric oxide release (47, 50). These results again illustrate that an increase in [Ca^2+^]_i_ by itself is not enough to activate the overt inflammatory actions of microglia and may even inhibit them. They also emphasize that overt inflammatory responses are not required for microglia to participate in pathological processes and nervous system disorders (Figure 2).

## Role of Voltage-Gated and Store-Operated Ca^2+^ Channels in Microglial Function

As discussed earlier, rat, mouse, and human microglia express a variety of Ca^2+^ channels, including voltage-gated Ca^2+^ channels and Ca^2+^-release-activated Ca^2+^ channels (1, 51). A study by Tokuhara et al. demonstrated involvement of N-type calcium channels in monocyte chemoattractant protein-1 (MCP-1) production from microglia. MCP-1 released from glial cells acts as a chemokine and attracts a variety of cells, including monocytes, T lymphocytes, and dendritic cells, to the brain (52). Store-operated Ca^2+^ entry (SOCE) has been demonstrated as another mechanism that supplies Ca^2+^ for intracellular processes in microglia via Orai1/I_CRAC_ channels (53). More recent evidence suggests that Ca^2+^ entry via SOCE plays an important role in responses to purines and BDNF and contributes to the activation of intracellular pathways causing cytokine secretion, phagocytosis, and chemotaxis (54, 55). In general, however, the role of these sources of Ca^2+^ in microglial signaling has not been investigated as thoroughly as have glutamate and purinergic receptors.

## Microglial Expression of K^+^ Channels – Kv1.3 and 1.5

In addition to the role of ligand-gated ion channels and calcium influx, many studies have also demonstrated a critical role of K^+^ channels in various pro-inflammatory microglial functions. This is presumably because K^+^ channels prevent depolarization of the membrane and maintain the driving force for Ca^2+^ entry as is proposed to occur in peripheral immune cells (56). The variety of these channels, however, and their roles in different aspects of microglial function have proven complex to unravel.

Initial reports suggested that, in culture, unstimulated microglia express only inward rectifying K^+^ channels (57), but that purine-, LPS-, gamma interferon-, or granulocyte macrophage colony stimulating factor-stimulated microglia display an outward K^+^ conductance (57–60). For instance, Norenberg et al. demonstrated a protein synthesis-dependent expression of outward K^+^ conductance peaking at 3 h after LPS exposure, which then gradually decreased despite the continued presence of LPS (57, 58). By contrast, Khanna et al. reported the presence both Kv1.5 and Kv1.3 channel protein in unstimulated microglial cell lysates, but saw only Kv1.3 like-current as demonstrated by the complete blockade of the voltage-dependant current by agitoxin-2. Immunohistochemical analysis demonstrated that Kv1.3 was present on the microglial cell membrane, whereas Kv1.5 was found mainly intracellularly (2). Another *in vitro* study demonstrated a functional role for Kv1.5 in microglia (61). Unlike control/wildtype microglia, LPS-stimulated production of nitric oxide was not seen in microglia isolated from Kv1.5^−/−^ knockout mice or microglia pre-treated with antisense oligonucleotide (AO) for Kv1.5, whereas LPS-stimulated chemokine release remained intact. By contrast, AO for Kv1.3 or pharmacological blockade of Kv1.3 did not inhibit LPS-stimulated NO production, suggesting that KV1.5, but not Kv1.3 is required for the microglial NO release function (61, 62). Pannasch et al. also reported that decreasing expression of either Kv1.5 or Kv1.3 channels can prevent the LPS-mediated decrease in microglial proliferation. Moreover, increased microglial proliferation was observed in Kv1.5^−/−^ mice after facial nerve injury *in vivo* (61). Collectively, these studies suggest that inflammatory stimuli increase expression of microglial Kv 1.5 channels, which is required for increased NO production and inhibition of the cell cycle and but not chemokine release.

As mentioned above, Kv1.3 channels do appear to have a role in microglial proliferation (3), as well as cytokine release (63), morphological changes, and the NADPH oxidase-dependent respiratory burst response to PMA (2) as demonstrated by pharmacological blockade. Adhesion has also been suggested to regulate both microglia responses and expression of microglial K^+^ channels. Integrins are transmembrane glycoproteins responsible for regulation of adhesion and the ability of immune cells to migrate. Microglia express several different integrin, including α4, α5, α6, β1, lymphocyte function-associated antigen-1, and Mac 1 β2-integrin. Interestingly, the pharmacological inhibition of Kv1.3 channels or of β-integrin also inhibits microglial migration toward various chemo attractants (64), although ATP was not tested. These results clearly indicate that Kv1.3 channels are also involved in microglial migration.

Fordyce et al. also demonstrated that blockers of Kv1.3 channels, but not Kv1.2, Kv1.5, and Kv1.6, inhibited the respiratory bust and consequent killing of neurons by microglia in co-culture (62), suggesting that Kv1.3 blockers may be useful as neuroprotective agents. With the development of potential therapeutic agents targeting this channel, their protective properties have been confirmed in a rat model of radiation-induced brain injury (65). The potential importance of these findings is emphasized by the recent demonstration of high levels of Kv1.3 in microglia in human brains from Alzheimer’s disease patients (66).

## Other K^+^ Channels with Roles in Microglial Cytokines, Nitric Oxide, and ROS Production

Some studies have also suggested a functional role for Kv1.1 and Kv1.2 shaker-like voltage-gated K^+^ channels in microglia. These channels are present in early postnatal microglia but disappear in ramified microglia present in adult rat brain. *In vitro* studies have reported induced expression (mRNA as well as protein) of Kv1.1 and Kv1.2 channels in microglia upon activation with LPS, hypoxia (67, 68), or ATP (68). Application of neutralizing antibodies to Kv1.1 is reported to reduce microglial cytokine (TNF-α, IL-1β) and nitric oxide production upon exposure to LPS or hypoxia (67). In contrast to Fordyce et al., Li et al. reported that an inhibitor of Kv1.2 channels reduced microglial cytokine mRNA production (TNF-α and IL1-β) and completely prevented ROS production in microglia and BV-2 cells exposed to LPS or hypoxia (68). Notably, none of the studies on Kv1.1 and Kv1.2 channels have reported complete inhibition of cytokine production suggesting the involvement of other pathways or ion channels.

Several studies have reported a functional role of Ca^2+^-activated K^+^ channels in microglial function. For instance, the respiratory burst, but not the morphological changes in response to PMA were shown to be dependent on the Ca^2+^/calmodulin-gated channels SK2, SK3, and SK4 (2). These investigators have now elaborated on this mechanism in a study published as part of this research topic (69).

## Conclusion

In conclusion, microglial activation occurs via a cascade of extracellular and intracellular signaling events beginning with cell surface receptor and ligand-gated ion channel activation followed by short-term and long-term changes in intracellular Ca^2+^. These Ca^2+^ signals can cause release of other factors in autocrine paracrine feedback loops and are necessary, although not sufficient for various subsequent cellular responses in microglia. The roles of various K^+^ channels in enabling the different subsequent pro-inflammatory responses of microglia have been clearly demonstrated. Their role in more subtle microglial responses, in particular “alternate activation” and P2X-mediated events require more careful investigation. These more subtle events are potentially just as clinically relevant as the more overt inflammatory functions of microglia and so a deeper understanding of the role of ion channels in these responses will no doubt have major consequences for human health.

## Conflict of Interest Statement

The authors declare that the research was conducted in the absence of any commercial or financial relationships that could be construed as a potential conflict of interest.
